# Folic Acid Supplementation Adherence During Pregnancy: Evidence from a Cross-Sectional Survey in Italy

**DOI:** 10.3390/nu18203332

**Published:** 2026-10-12

**Authors:** Francesca Licata, Concetta Arianna Scicchitano, Lucrezia Amodei, Aida Bianco

**Affiliations:** Department of Medical and Surgical Sciences, School of Medicine, University of Catanzaro “Magna Gracia”, Viale Europa, 88100 Catanzaro, Italy; f.licata@unicz.it (F.L.); a.scicchitano@unicz.it (C.A.S.); lucreziaamodei@hotmail.it (L.A.)

**Keywords:** folic acid, neural tube defects, planned pregnancy, pregnancy, pregnant women, prevention

## Abstract

**Background/Objectives**: Periconceptional folic acid (FA) supplementation is a cornerstone of primary prevention of neural tube defects (NTDs). However, adequate preconceptional FA intake remains suboptimal in Italy. This study aimed to assess FA use among pregnant women in the Calabria region, determine whether their intake met United States Preventive Services Task Force (USPSTF) recommendations, and explore potential predictors that shape these practices. **Methods**: This cross-sectional study was conducted among pregnant women using face-to-face interviews. The adequacy of FA intake was based on the USPSTF guidelines, which recommend a daily FA intake of 400–800 μg, starting at least one month before conception and continuing through the first two to three months of pregnancy. **Results**: Among 421 women, 92.3% reported FA intake, predominantly following a medical prescription (86.3%), usually by a gynecologist rather than a general practitioner. General awareness of FA was notably high (79.3%), whereas preconceptional adherence was strikingly low (25.8%). Indeed, among women with planned pregnancies, only 22.6% achieved adequate intake. Furthermore, the strongest predictors of preconception FA use were having received a healthcare worker’s recommendation (aOR: 26.84; 95% CI: 11.40–63.17) and having a planned pregnancy (aOR: 6.03; 95% CI: 1.92–18.90). **Conclusions**: Despite widespread general knowledge, timely preconceptional FA use remains low, reflecting a “knowing–doing” gap and an over-reliance on specialist-driven prescription. These findings support integrating FA counseling into routine primary care and considering mandatory food fortification to ensure equitable, population-level prevention of NTDs, independent of women’s education status or pregnancy intention.

## 1. Introduction

Neural tube defects (NTDs) are a heterogeneous group of congenital malformations, encompassing conditions such as anencephaly, spina bifida, encephalocele, craniorachischisis, and iniencephaly [1]. NTDs represent a major global health concern associated with high morbidity and mortality. Furthermore, they have significant social and health impacts, as even non-lethal forms often require complex surgical interventions, followed by prolonged and costly rehabilitation pathways that may not always be curative. As such, the impact of NTDs extends beyond the affected individual, placing a substantial burden on families and healthcare systems alike [1,2,3]. In Europe, birth prevalence estimates range from 0.94 to 69.6 cases per 10,000 live births. In Italy, data from the Italian Ministry of Health indicate an NTD prevalence of approximately 6 cases per 10,000 live births, with spina bifida accounting for nearly 50% of these cases [4].

Maternal folic acid (FA) supplementation is one of the most effective preventive interventions for reducing the risk of NTD, with optimal benefits achieved when initiated before conception and continued through the first two to three months of pregnancy. The effects of FA have been extensively studied in relation to NTDs and fetal growth, as folate deficiency at conception and during pregnancy can lead to abnormal fetal development [1]. Evidence consistently demonstrates that adequate FA intake reduces the risk of NTDs by 50–70% [5,6]. Beyond NTDs, adequate FA intake has been associated with a reduced risk of other congenital anomalies, including congenital heart defects and limb malformations, as well as protection against maternal complications like megaloblastic anemia and peripheral neuropathy [7,8]. Major international guidelines, including the 2023 update from the United States Preventive Services Task Force (USPSTF), recommend that all women planning or capable of becoming pregnant should initiate daily FA supplementation of 400 to 800 μg of FA at least 1 month prior to anticipated conception and continue through the first 2 to 3 months of pregnancy [9]. These recommendations align with those of other international health organizations, such as the World Health Organization (WHO) and the Centers for Disease Control and Prevention, which emphasize the importance of FA supplementation for the primary prevention of NTDs [10,11]. In Italy, adherence to these recommendations remains unsatisfactory [12]; adequate intake exhibits significant geographic variability, ranging from 24.6% of women in Campania to 44.4% in Veneto, with consistently higher rates observed in the northern regions compared to the south [12]. Given the established importance of FA throughout pregnancy, the primary objective of the present study was to characterize FA utilization patterns and evaluate their correlation with participants’ characteristics and knowledge. Additionally, the secondary objective was to assess the adequacy of FA intake.

## 2. Materials and Methods

### 2.1. Study Design and Setting

The present cross-sectional study was conducted between April and September 2023 among a sample of pregnant women of the Calabria region, Southern Italy. The study reporting complies with the Strengthening the Reporting of Observational Studies in Epidemiology (STROBE) guidelines [13].

### 2.2. Study Population and Data Collection

The inclusion criteria for participation in this study were as follows: (i) being aged 18 years or older (ii) being able to understand the Italian language, and (iii) being pregnant at any gestational age.

Based on these eligibility criteria, a multi-stage sampling design was employed. In the first stage, women’s health clinics and Departments of Obstetrics and Gynecology at general hospitals were selected through simple random sampling across the three geographical areas of the region (north, central, and south). The study objectives and methodology were explained to the facility staff prior to obtaining authorization for the survey. All invited facilities agreed to participate. The target population comprised consecutive pregnant women attending the outpatient women’s health clinic or admitted to the Departments of Obstetrics and Gynecology on randomly selected days.

Before data collection commenced, all participants were informed about the background, objectives, and privacy policies of the survey. Written informed consent was obtained from all individuals who agreed to participate in the study, clarifying that the anonymity and confidentiality of the collected data were guaranteed. After obtaining written informed consent, the research team conducted a face-to-face interview. No payment or incentives were offered to participants for taking part in this study.

### 2.3. Questionnaire Design

The questionnaire was developed following an extensive literature review [14,15,16] and was subsequently pretested on a small pilot group of 10 eligible pregnant women who were excluded from the final sample. Based on the feedback received during this phase, minor refinements were made. The final questionnaire took approximately 15 min to complete and consisted of 22 questions grouped into 3 sections. The first section gathered participants’ sociodemographic (i.e., age, educational level, number of children in the household, marital status, and employment status) and obstetric history (i.e., gestational age, parity, pregnancy-related conditions, whether the pregnancy was planned, and perceived health status during pregnancy) characteristics. The second section investigated general knowledge regarding FA supplementation and its importance during pregnancy; moreover, it also explored the self-perceived need to receive additional information about FA intake. The final section examined the actual behaviours toward recommended FA intake (i.e., use during the preconceptional period and throughout pregnancy, dosages, as well as the reasons for taking it and the source of the recommendation). Ethical approval for this study was granted by the Ethics Committee of the Central Area of the Calabria Region (ID No.275/2021/07/15).

### 2.4. Adequate Folic Acid Intake

Adequate FA intake among women with planned pregnancy was defined in accordance with the USPSTF guidelines based on three specific criteria: (1) initiation of FA supplementation at least 1 month prior to anticipated conception for all persons planning to or who are capable of becoming pregnant (2) continued supplementation through the first 2 to 3 months of pregnancy, and (3) adherence to the recommended daily dosage of 400 μg to 800 μg of FA [9].

### 2.5. Statistical Analysis

Continuous variables were summarized using means and standard deviations (SD) when normally distributed, whereas medians and interquartile ranges (IQRs) were used in cases of non-normal distribution. The normality of the variables was assessed using the Shapiro–Wilk test. Categorical variables were expressed as frequencies and percentages.

A logistic regression model was performed to evaluate the relationship between each independent variable and the outcome of interest, defined as having initiated FA supplementation during the preconception period (i.e., ≥4 weeks before conception). The logistic regression model was developed according to the Hosmer and Lemeshow strategy [17], and independent variables with a *p*-value ≤ 0.25 in the bivariate analysis were included in the multivariable model. Epidemiologically relevant variables were also included in the model, regardless of the results of the bivariate analysis; the rationale for this approach was to provide as complete a control of confounding factors as possible within the given dataset. The model was built to determine the explanatory variables independently related to the dichotomous measure of having started the FA supplementation in the preconception period (Model 1). The following independent variables were included in the model: age (in years, continuous), education level (high secondary school or lower = 0, university degree = 1), having at least one chronic illness (no = 0, yes = 1), marital status (married/cohabiting with a partner = 0, single/divorced = 1), occupational status (unemployed = 0; employed = 1), having one or more children (no = 0, yes = 1), having received a recommendation from a Health Care Worker (HCW) to begin FA intake before conception (no = 0; yes = 1), having a planned pregnancy (no = 0; yes = 1), and knowledge regarding FA and the purpose of its intake (no = 0; yes = 1). The statistical significance level was set at a *p*-value < 0.05. Adjusted odds ratios (aORs) and 95% confidence intervals (CIs) were calculated. Data were analyzed using STATA software, version 19 [18].

## 3. Results

### 3.1. Sociodemographic and Obstetric Characteristics

Table 1 presents the sociodemographic and obstetric characteristics of the participants. The study sample consisted of 421 pregnant women, with a mean age of 31.4 years (SD ± 5.04). Most participants (90.3%) were married or cohabiting, and approximately two-thirds had completed up to a high school education (61.2%) and were employed (60.8%).

Regarding gestational age at enrollment, the vast majority (85.3%) of the sample was in the third trimester, followed by 13.3% in the second and 1.4% in the first trimester. Among the respondents, 29.9% had only one child, while 18.1% had two or more children.

The majority of participants (76.7%) reported a planned pregnancy. A high-risk pregnancy due to preexisting maternal chronic medical conditions was reported by 5.2% of the respondents and due to pregnancy-related conditions by 7.1%. When asked about their perceived health status during the current pregnancy, 92.6% of the women reported it as good health status.

### 3.2. Knowledge Regarding Folic Acid Supplementation

Table 2 presents the participants’ responses regarding their knowledge of FA supplementation. The majority of the sample (79.3%) correctly identified FA as a vitamin, whereas only 9.5% reported having no knowledge of it. Furthermore, 87.2% of the women correctly identified its role in reducing the risk of NTDs and treating anemia; however, more than half (65.3%) expressed a need for additional information regarding FA supplementation.

### 3.3. FA Utilization Patterns and Adequacy

Table 3 presents the FA utilization pattern and dosage regimen. Less than half (47.8%) of the sample received advice from HCWs to initiate FA supplementation before becoming pregnant. Overall, 388 women (92.2%) reported FA intake, with the majority (86.3%) initiating supplementation following a medical prescription. Among these, over half were advised by their gynecologist (63.1%), whereas a significantly smaller proportion (12.1%) obtained their prescription from a general practitioner (GP). Additionally, 12.6% of participants reported initiating FA intake on their own initiative. Regarding the timing of FA supplementation, only a minority of women (25.8%) initiated intake preconceptionally (i.e., ≥4 weeks before conception). Furthermore, just 2.1% limited their FA use to the first trimester, with the vast majority (97.9%) continuing supplementation throughout the entire pregnancy and into the postpartum period. Regarding the dosage regimen, 97.1% of the women took 400 μg to 800 μg per day. Among planned pregnancies, the overall adequacy of FA intake was 22.6%. All 87 women in this subgroup initiated FA supplementation at least one month prior to conception, and 96% of them continued intake throughout the first 2–3 months and until the end of pregnancy. Regarding dosage, 83.6% of these women took 400–800 μg per day. As shown in Model 1 (Table 4), having received a recommendation from a HCW to begin FA supplementation before conception (aOR: 26.84; 95% CI: 11.40–63.17) and having a planned pregnancy (aOR: 6.03; 95% CI: 1.92–18.90) were the strongest predictors of preconception FA utilization. Additionally, for every one-year increase in age (aOR: 1.09; 95% CI: 1.02–1.17), the odds of having initiated FA supplementation during the preconception period increased by 9%. Furthermore, pregnant women with a university degree (aOR: 2.60; 95% CI: 1.35–5.00) were more likely to have initiated FA supplementation during the preconception period.

## 4. Discussion

By detailing the knowledge and utilization patterns of FA among pregnant women, this study exposes a striking public health paradox. While recent Italian data indicate an impressive overall FA uptake—with approximately 90% of women reporting supplementation—only a marginal fraction adheres to the critical preconception window necessary to effectively prevent NTDs [12]. Ultimately, our findings underscore that high overall consumption masks a severe timing deficit, offering actionable evidence to redesign targeted public health strategies.

A key finding of our study is the stark discrepancy between the high level of general knowledge regarding FA (79.3%) and the alarmingly low rate of preconceptional adherence (25.8%). This phenomenon, often described as the “knowing–doing” paradox, is consistently mirrored in the international literature. Global systematic reviews evaluating FA supplementation trends indicate that while past public health campaigns have successfully educated women of childbearing age about the benefits of FA in preventing NTDs, this theoretical awareness rarely translates into timely, preconceptional intake [19]. For instance, studies conducted across various European and North American populations demonstrate that although maternal awareness of FA often exceeds 70%, actual preconceptional utilization persistently stagnates between 20% and 40% [20,21]. This evidence suggests that current global public health paradigms, which have predominantly focused on the “what” and “why” of FA supplementation, have reached a ceiling effect. To bridge this critical gap, future health literacy interventions must shift their primary focus from basic awareness to the crucial “when,” aggressively emphasizing the narrow, early developmental window during which supplementation is effective. In this context, our data reveal that a substantial proportion of participants (74.2%) initiated FA supplementation exclusively following pregnancy confirmation. Consequently, for these individuals, the critical embryological window for neural tube closure, which occurs between gestational days 21 and 28 [22], often before pregnancy is even recognized, had already elapsed prior to the first administered dose. This delayed initiation indicates that the primary barrier to effective primary prevention is no longer a deficit in general awareness regarding FA utility but rather a specific lack of health literacy concerning the strictly time-dependent nature of preconceptional supplementation.

Although the vast majority of women who took FA received a medical prescription, these were predominantly issued by gynecologists rather than GPs. This over-reliance on specialist care represents a systemic flaw observed in other international healthcare settings [23]. Evidence suggests that women typically schedule gynecological or obstetric appointments only after a pregnancy is suspected or confirmed, by which time the narrow developmental window for neural tube closure has often already passed [24]. Consequently, delegating primary prevention to specialists inherently delays the initiation of FA supplementation. To overcome this barrier, international public health experts increasingly advocate for the integration of preconception counseling into routine primary care [25]. Because GPs and family planning centers routinely interact with women of reproductive age for general health needs, they are uniquely positioned to proactively deliver FA counseling long before conception occurs, thereby shifting the clinical paradigm from reactive to truly preventive care. These findings strongly underscore the necessity of systematically integrating structured preconception counseling within primary care frameworks. To optimize preventive outcomes, such counseling must transcend general recommendations on FA efficacy and provide explicit clinical directives regarding the precise timing of supplementation. Specifically, healthcare providers must emphasize that initiating FA intake upon pregnancy recognition is temporally insufficient for NTD prevention and that the prophylactic window rigorously mandates initiation at least four weeks prior to anticipated conception.

A critical implication of this study arises from the strikingly low adherence rate (22.6%) to USPSTF guidelines, even among women with planned pregnancies. This finding exposes the fundamental inadequacy of relying exclusively on voluntary, behaviour -dependent supplementation strategies. International epidemiological data corroborate this, demonstrating that public health policies based solely on education and voluntary pill consumption consistently fail to achieve the population-level coverage required for optimal NTD prevention [26]. Undoubtedly, an appropriate and balanced diet rich in natural folates—found in leafy green vegetables, legumes, and citrus fruits—must always constitute the foundation of maternal health and represent the primary source of overall nutrients. However, in the specific context of primary prevention, diet alone has intrinsic limits due to differences in bioavailability. Because only about 50% of folate naturally present in food is bioavailable compared to the near 100% absorption of synthetic supplements, dietary intake alone—even in a perfectly balanced diet—is widely considered insufficient to achieve the optimal red blood cell folate concentrations strictly required to prevent NTDs and other adverse outcomes associated with insufficient folate intake during the periconceptional period [27]. Therefore, while diet provides essential baseline nutrition, strict clinical adherence to synthetic FA supplementation remains an irreplaceable primary intervention, especially since dietary habits may remain unchanged even among couples planning a pregnancy [26,28]. Consequently, our results contribute to the ongoing global debate, highlighting the potential value of considering FA fortification of staple foods as an equitable, passive public health intervention that increases the amount of bioavailable folate in the diet of the whole population, ensuring a baseline intake regardless of individual behaviour. Such programs have successfully reduced NTD prevalence in over 80 countries, including the United States and Canada [1]. While many European countries have historically hesitated to adopt widespread fortification policies—often relying on the protective effects of traditional nutritional patterns, such as the Mediterranean diet—the progressive globalization of food systems and the rapid erosion of these dietary habits suggest that relying solely on diet may become increasingly challenging [28]. In women of childbearing age, fortification should complement rather than replace supplementation, and international guidelines recommend obtaining 400 μg/day of FA from dietary supplements and/or fortified foods in addition to the folate provided by a varied diet. Transitioning toward mandatory fortification policies thus emerges as a public health imperative to eliminate preventable NTDs, effectively bypassing the intrinsic limitations of individual adherence and structural counseling barriers. Investing in such preventive strategies is essential as, in the European Union, over 4500 pregnancies are affected by NTDs each year, with no decline in prevalence over the past three decades [29]. Beyond their clinical burden, NTDs entail substantial and lifelong costs for healthcare systems and families. In Italy, a cost-of-illness study estimated an annual cost of approximately €11,350 per patient with spina bifida, mostly attributable to assistive devices and non-healthcare costs, corresponding to an overall social cost of about €60 million per year [30]. Hence, improving timely FA supplementation and considering population-level measures such as food fortification may represent not only an effective but also an economically advantageous public health strategy. 

Our analysis reveals a pronounced sociodemographic gradient in FA utilization, with younger maternal age and lower educational attainment acting as significant barriers to preconception adherence. This inequitable distribution of preventive care is a well-documented challenge in international maternal health research [31]. When public health systems rely exclusively on voluntary, prescription-based supplementation, they inadvertently exacerbate existing health disparities [32]. Specifically, such individualistic strategies disproportionately benefit older, highly educated women who possess the health literacy and socioeconomic resources to proactively seek preconception counseling, while leaving marginalized or younger populations largely unprotected. Consequently, addressing this social gradient requires shifting the focus from individual compliance to systemic equity. To achieve this, culturally tailored educational interventions must be coupled with universal public health policies, such as mandatory food fortification, that guarantee equal protection against NTDs regardless of a woman’s socioeconomic status, educational background, or pregnancy intention [32,33].

Future research should adopt a multidimensional approach to evaluate maternal folate status comprehensively. To accurately estimate total folate intake, self-reported supplementation data should be integrated with validated dietary assessment tools, such as food frequency questionnaires or 24-h recalls. Concurrently, incorporating objective blood biomarkers (e.g., serum or red blood cell folate and homocysteine concentrations) and genetic profiling—particularly MTHFR genotyping—is crucial to explore interindividual differences in folate metabolism and baseline requirements [34]. Integrating these nutritional, biochemical, and genetic parameters could pave the way for individualized periconceptional care. As demonstrated by the clinical practice guidelines of the Society of Obstetricians and Gynaecologists of Canada [35], which advocate for targeted folate screening in high-risk groups (e.g., women taking medications with antifolate effects), a precision-medicine approach is increasingly necessary to inform tailored dose adjustments. Ultimately, alongside these clinical advancements, cost-effectiveness analyses must be performed to determine whether incorporating such comprehensive dietary assessments and biomarker screenings into routine preconception care pathways provides tangible public health benefits beyond the current standard of universal supplementation.

### Limitations

When interpreting the study’s findings, several limitations should be acknowledged. First, one limitation is the study’s cross-sectional design, which does not allow for causal conclusions regarding the observed associations. Nevertheless, this study can describe general associations. Second, relying on self-reported data without biochemical validation introduces potential recall and social desirability biases. However, we expect the impact of both to be marginal: strict anonymity protected participants against social pressure, while limiting the timeframe to the current pregnancy minimized memory-related inaccuracies. Another limitation is the potential overestimation of positive outcomes, as women who were more informed or interested in health topics may have been more willing to participate. If anything, this suggests that the true rates of adequate preconceptional FA supplementation in the general pregnant population of Calabria may be even lower than those observed here, further strengthening rather than undermining the public health concern raised by our findings. Fourth, the study data were collected from pregnant women from only one Italian region, which may not represent the entire pregnant population in Italy. However, it is reasonable to suppose that the study’s results are at least representative of pregnant women in southern Italy. Fifth, this study lacked data on dietary folate intake (e.g., 24-h recalls or food diaries), precluding the estimation of total folate intake or dietary confounders. However, our primary aim was to assess adherence to universal clinical guidelines (e.g., USPSTF, WHO), which mandate 400–800 µg/day of FA regardless of maternal diet. This universal mandate is dictated by pharmacokinetics, given the marked differences in bioavailability between synthetic FA and natural food folates [27], as detailed above.

## 5. Conclusions

In conclusion, while our findings originate from a regional context, they expose a universal public health challenge: high awareness of FA does not naturally translate into timely, effective prevention. The persistent failure to achieve adequate periconceptional adherence—even among women with planned pregnancies—underscores the inherent limitations of relying on voluntary, specialist-driven supplementation, a strategy that inadvertently widens sociodemographic health disparities. To effectively eliminate the global burden of NTDs, a paradigm shift is urgently required. Public health frameworks must transition from merely educating patients to systematically empowering primary care networks to deliver early, proactive counseling. Concurrently, these findings strongly advocate for the adoption of equitable, population-level interventions, such as mandatory food fortification. Ultimately, bridging the critical gap between knowledge and action demands systemic solutions that guarantee protection for all women, irrespective of their socioeconomic status, health literacy, or pregnancy intentions.

## Figures and Tables

**Table 1:** General characteristics of FA use during pregnancy among women in the Calabria region.

Variables	*n* (*n* = 421)	**%**
**Age (mean, SD** **±)**	31.4	±5.04
**Marital status**		
Married/Cohabitant	380	90.3
Single/Divorced/Separated	41	9.7
**Educational level**		
Elementary school/Secondary school/High School	257	61.2
University degree	64	38.8
**Occupation**		
Employed	256	60.8
Unemployed	165	39.2
**Number of previous births**		
None	219	52
1 birth	126	29.9
≥2 births	76	18.1
**Gestational age at enrollment**		
First trimester (1–13 weeks)	6	1.4
Second trimester (14–26 weeks)	54	13.3
Third trimester (27–40 weeks)	345	85.3
**Planned pregnancy**		
Yes	323	76.7
No	73	17.4
**High-risk pregnancy**		
No	369	87.7
Yes, for preexisting chronic medical condition	22	5.2
Yes, for pregnancy-related morbidity	30	7.1
**Perceived Health Status**		
Good	386	92.6
Poor	31	7.4

Total may not always sum to *n* because of missing data. FA: Folic Acid.

**Table 2:** Knowledge about FA supplementation.

Variables	*n* (*n* = 421)	%
**FA is**		
Hormone	47	11.2
** Vitamin**	**334**	**79.3**
I do not know	40	9.5
**Purpose of taking FA during pregnancy** ^#^		
** To prevent NTD (e.g., spina bifida)**	**352**	**83.6**
To prevent gestational nausea	43	10.2
** To treat anemia**	**35**	**6.3**
To prevent ocular malformations	112	20
I do not know	7	1.3
Other	2	1.4
**Need for further information on FA supplementation**		
Yes	275	65.3
No	144	34.4

Total may not always sum to *n* because of missing data. ^#^ Multiple responses allowed. Correct answers are marked in bold. FA: Folic Acid; NTD: Neural Tube Defect.

**Table 3:** FA pattern uses and dose regimen.

Variables	*n* (*n* = 421)	%
**Being advised to start taking FA before becoming pregnant by HWCs**		
Yes	194	47.8
No	202	49.6
I do not know	10	2.6
**FA supplementation**		
Yes	388	92.2
No	33	7.8
**Preconception supplementation of FA**		
Yes	100	25.8
No	288	74.2
**Duration of FA intake during pregnancy**		
Through the first 2 to 3 months of pregnancy	8	2.1
Until the end of the pregnancy	378	97.9
**Daily dose of FA supplementation (μg/day)**		
400–800 μg	332	97.1
≥800 μg	10	2.9

Total may not always sum to *n* because of missing data. FA: Folic Acid; HWCs: Health Care Workers.

**Table 4:** Results of the logistic regression model of potential determinants of preconception FA supplementation among pregnant women.

**Outcome: having initiated FA supplementation in the preconception period** *Log* *likelihood = −142.00503; Prob > chi2 < 0.0000; Obs = 368*			
**Variables**	**aOR**	**95% CI**	***p-*** **Value**
**Having received a recommendation from an HCW to start taking FA before conception**			
No *	1.00		
Yes	26.84	11.40–63.17	<0.001
**Planned pregnancy**			
No *			
Yes	6.03	1.92–18.90	0.002
**Educational level**			
High secondary school or lower *	1.00		
University degree	2.60	1.35–5.00	0.004
**Age in years, continuous**	1.09	1.02–1.17	0.016
**Marital status**			
Married/cohabiting with a partner *	1.00		
Single/divorced	0.55	0.09–3.19	0.501
**Occupational status**			
Unemployed *	1.00		
Employed	0.81	0.40–1.64	0.564
**Having at least one chronic illness**			
No *	1.00		
Yes	1.17	0.60–2.31	0.644
**Having more than one child**			
No *	1.00		
Yes	1.12	0.61–2.06	0.721
**Correct knowledge and purpose of FA**			
No *	1.00		
Yes	1.05	0.48–2.30	0.911

* Reference category. 95% CI: 95% Confidence Interval; aOR: Adjusted Odds Ratio; FA: Folic Acid; HWC: Health Care Worker.

## Data Availability

The original data presented in this study are openly available in the Mendeley Data repository (https://doi.org/10.17632/k9ck73bt3d.1).

## Abbreviations

The following abbreviations are used in this manuscript:

95% CIs	95% confidence intervals
aORs	Adjusted Odds Ratios
FA	Folic acid
GP	General practitioner
WHO	World Health Organization
HCW	Health Care Worker
IQRs	Interquartile Ranges
NTDs	Neural tube defects
SD	Standard Deviation
USPSTF	United States Preventive Services Task Force
